# Editorial: facts, figures and the future

**DOI:** 10.1186/s13049-014-0079-6

**Published:** 2015-01-09

**Authors:** Kjetil Søreide

**Affiliations:** Department of Gastrointestinal Surgery, Stavanger University Hospital, Stavanger, Norway; Department of Clinical Medicine, University of Bergen, Bergen, Norway

Since the name-change to its current title over 2 decades ago [1], the *Journal* has seen remarkable development and progress. After hard work, the *Journal* was accepted and indexed in PubMed in 2008 as an open access journal [2], a factor that is perceived to be important to the journal prosperity [3]. Being accepted by Thomson Scientific and the Web of Science indexing system, the Journal got the inaugural impact factor (IF) in 2010. Landing a first IF at a staggering 2.18 was remarkable indeed and placed the journal among the top 3 world leading journals in the category “emergency medicine” that year. The slight decrease in IF over the two proceeding years was in many ways anticipated – the number of papers accepted and published has gradually increased, levelling off at about 80–90 papers per year. Notably, increasing the denominator negatively influences the IF in relation to number of citations. However, we are confident that this will again turn to the advantage of the journal as we now see a sound inflow of manuscripts at the same time as the number of citations is steadily increasing year on year. It is thus comforting to see a turn on the IF at 1.93 this year. While not quite back at the IF above 2-level (Figure 1), we aim at achieving this goal in the near future.

**Figure 1:**
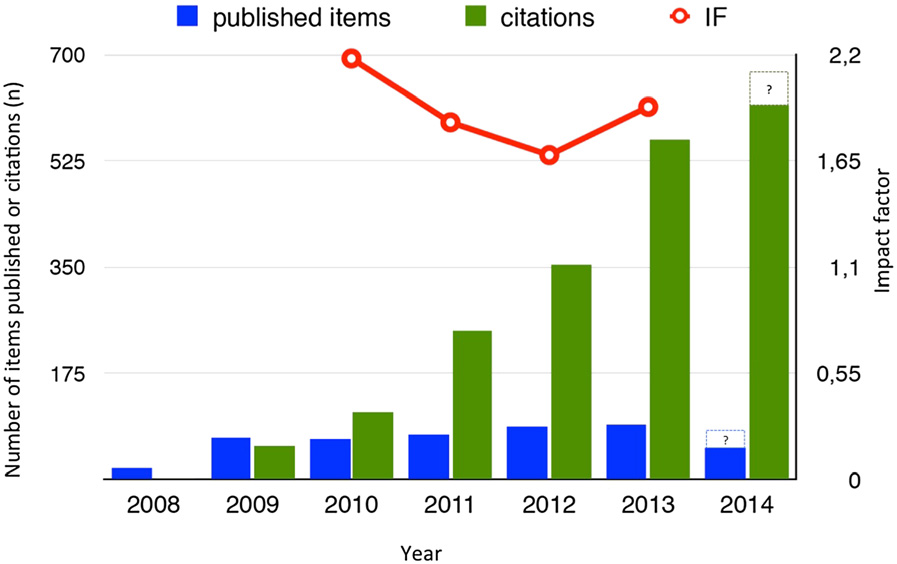
**Depiction of published items, citations per year, and the corresponding impact factor.** Note that the collection of published papers and citations is not complete for year 2014. Please note that not all publishable items are counted as citable items.

With a slight decline in accepted manuscript for 2014, largely due to a more restrictive policy on case reports, we anticipate the firm inflow of good papers to contribute to this achievement. Acceptance rate is still close to 40%, with some 12% of accepted papers being systematic reviews and meta-analyses [4-9], and the gross volume original research papers covering a wide range of research methodology. The Journal continues to support consensus reports [10-12], and remains the vehicle for abstracts from national and international meetings [13-15]. Randomized design and multicentre studies are increasingly accepted [16-19], as well as the protocols for future planned and ongoing studies [16,20,21]. Indeed, SJTREM has also published animal studies [22-24] and papers including biomarkers and genomic technology [25,26], which points to the diversity with which we are exposed.

Importantly, IF is not the only metric that may reflect a journal’s value. Readers may be interested to know that the journal H-index is 17, meaning that 17 papers are cited 17 times and more, which is rather good considering the ‘young age’ of the journal. At the time of writing, almost 2000 accumulated citations to SJTREM papers have occurred, with the majority in the latter period (Figure 1). The top 3 cited papers of all time are still accumulating an annual average number of citations of 17, 12 and 8 cites per year [27-29], which is impressive and demonstrates longevity in the value of these papers. Also, all of the top 10 cited papers continue to have average citations per year in the range of 4 to 17. The new publishing platform on SpringerOpen [30] indicates short of 800 manuscript units published in SJTREM since 2008, of which short of 500 are now indexed in Thomson Scientific (not counting in the lag-time from accept, to publishing, to PubMed and WoS indexing). This points to a sound and steady inflow of manuscripts.

The relatedness to the content in SJTREM is also reflected in the number of citations received by other journals in the field. The journals that most frequently cited material published in SJTREM was (in ranking order; counting only journals with >10 citations) *Resuscitation*, *Emergency Medicine Journal*, *Journal of Trauma Acute Care Surgery*, *Injury*, *Transfusion*, *Critical Care*, *American Journal of Emergency Medicine* and *Acta Anaesthesiologica Scandinavia*. To the editors this is a testimony that material published in SJTREM is of interest to readership and investigators in the immediately related fields and, most importantly, receives attention outside Scandinavia and is cited both by European and North American based journals. We anticipate the attention and interest to increase with the growth and international perusal of journal content.

Moreover, we need to recognize that the world-wide readership also value other papers differently in terms of views and downloads (see Figure 2), which does not necessarily overlap with the same papers that are most highly cited. However, most of these papers are recent releases, and it is expected that they will accumulate citations over time. Indeed, the top-viewed paper by Haddad *et al.* [31] is among the most cited papers over the past 2 recent years in the journal. The difference in the lists of ‘most cited’ and ‘most viewed’ only reflects the diverse role of publishing and the spread between clinical information, educational material and development of research instruments. Not incorporated into this equation is the role of social media – we currently have no direct overview of tweets and blogs that spread the information published in SJTREM, but expect this to be considerable with the current distribution and use of smartphones and tablets worldwide.

**Figure 2:**
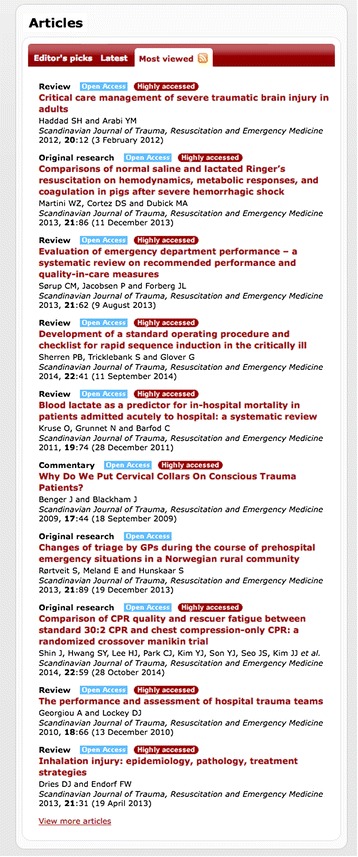
**Most viewed papers in SJTREM.** List of ‘most viewed’ papers in the Journal, status per December 2014. Screendump from the homepage www.sjtrem.com.

Behind the journal’s success is not only the continued support by the editorial team, but first and foremost the support by reviewers and the increased interest by researchers and readers of the *Journal*. The editorial policy has been developed along the way [32,33], yet the aims of serving as a multidisciplinary journal for emergency research in Scandinavia, Europe and internationally stands firm. We are happy to have Julian Thompson MD from the London’s Air Ambulance onboard as an associate editor, which strengthens our foothold in the pre-hospital part of the emergency disciplines. We also welcome as associate editors drs. Martin F. Kurz MD, PhD, consultant neurologist, and; Knut Øymar MD PhD, consultant and professor in paediatric medicine, both at the Stavanger University Hospital. We are positive that this will help us grow the spectrum of topics within acute and critical neurological illness as well as acute and critical paediatric disease; two areas where both innovation, research and progress have seen remarkable effects for patients. We are excited to see the development of this in the future Scandinavian and international perspective as well.

Lastly, it is hoped that the journal will receive manuscripts for evaluation from the field of emergency surgery to a greater extent. With about 245 million operations being performed each year globally, of which many if not most are emergent in nature, there is considerable potential in addressing surgical emergency conditions for human health [34], in both Scandinavia and beyond. Many urgent conditions experience mortality in the range of 20–30% and above – and may in some geographical areas far outweigh the burden of trauma and similar disease [35]. Planning, conducting and reporting research in emergency medicine and surgery may be a challenge indeed, also from an ethical, legal and practical point of view [36-39]. However, it should be stated that care of the acute or critically ill patients should not be seen in isolation, as cross-disciplinary work is needed to strengthen the chain of survival for all emergencies [40].

## Abbreviations

IF: Impact factor
SJTREM: Scandinavina Journal of Trauma Resuscitation and Emergency Medicine
MD: Medical doctor
PhD: Philosophiae doctor
H-index: Hirsch-index
WoS: Web of Science
